# Temporal structure of the junior badminton championship across all five events

**DOI:** 10.3389/fspor.2025.1710043

**Published:** 2026-01-05

**Authors:** Algislayne Fatchner Coenga, Lara Morgado da Silva Santos, Fabio Prado Galvão Machado, Tatiane Mazzardo, David Cabello-Manrique, Gibson Moreira Praça, Schelyne Ribas, Leyza Elmeri Baldo Dorini, Layla Maria Campos Aburachid

**Affiliations:** 1 Faculty of Physical Education, Federal University of Mato Grosso, Cuiabá, Brazil; 2 Departament of Informatics, Federal University of Technology of Paraná, Curitiba, Brazil; 3 Departament of Physical Education and Sports, Faculty of Sports Science, iMUDS, University of Granada, Granada, Spain; 4 Faculty of Physical Education, Physiotherapy, and Occupational Therapy, Federal University of Minas Gerais, Belo Horizonte, Brazil

**Keywords:** athletes, badminton, championship, game structure, match analysis

## Abstract

**Introduction:**

Understanding match demands in junior badminton is essential for designing training programs that support long-term athlete development. While previous research has focused on elite adult players, limited information is available on event-specific characteristics at the junior level.

**Methods:**

This study analyzed the temporal structure of matches from the 28th Pan-American Junior Badminton Championships, including men's single (MS), women's single (WS), men's double (MD), women's double (WD), and mixed double (XD) using observational data. A total of 124 matches were analyzed using video-based notational analysis. Key variables included match duration, real time played, % of real time played, rally time, rest time per rally, total points played, shots per rally, shot frequency, and work density.

**Results:**

The results showed that temporal structure is different between events, except in rest time per rally. Singles junior matches (MS and WS) are characterized by longer durations and rallies, whereas doubles matches (MD, WD, XD) involve higher shot frequency, shorter rallies, and more concentrated activity. There are clear event-specific profiles already present in junior as they are in elite-level competitions.

**Discussion:**

These findings support the need for event-specific training in junior badminton and highlight the importance for coaches and federations to gradually develop the physical and technical abilities of their athletes to meet elite performance demands.

## 1 Introduction

Badminton is one of the fastest racket sports worldwide, characterized by intermittent high-intensity efforts combined with short recovery periods. This sport demands a complex interaction of technical skills, tactical decision-making, and physical performance (1, 2). It is played across five events: men's singles (MS), women's singles (WS), men's doubles (MD), women's doubles (WD), and mixed doubles (XD), each presenting distinct match dynamics influenced by the number of players, sex, and tactical approaches.

In recent years, performance analysis in badminton has increasingly focused on elite competitions, investigating variables such as match duration (3), rally time (4), rest intervals (5), shot frequency (6), and technical–tactical actions (7). These studies have contributed to a better understanding of sport's physical and strategic demands (3, 5).

However, most research has focused on adult players and singles events, particularly men's and women's singles (5, 8, 9). Fewer studies have investigated double events (4, 10), and comprehensive analyses that include all five disciplines remain limited (11–15).

Previous studies often rely on small samples (14, 16) or do not consistently include all five events (4), which limits the generalizability of their findings. For example, Le Mansec et al. (14) observed that, among the women, doubles matches featured longer rallies, shorter intervals, and greater effective playing time, indicating that the differences relative to singles matches are less pronounced than in the men's matches.

As a result, applying these data to practical contexts such as talent identification, training design, and long-term athlete development remains challenging (11, 15). Although recent research has updated time structure data for elite adult competitions under the current rally-point scoring system (17), systematic analyses of junior competitions remain scarce, particularly in international tournaments that gather high-level youth athletes.

To date, only four studies have investigated junior singles matches in the U15–U19 age categories (16, 18–20). However, three of them were conducted in simulated match contexts (18–20), with the latter analyzing physiological variables, which limits its applicability to real competition scenarios.

In this context, the dependent variables analyzed in the present study include key temporal indicators essential for characterizing match demands in badminton, such as match duration, real time played, % of real time played, rally time, rest time per rally, total points played, shots per rally, shot frequency, and work density. These metrics provide insights into the intensity, pace, and technical–tactical complexity of each event, offering objective parameters to compare events. Systematic measurement of these indicators is crucial for guiding training design, adjusting physical and technical loads, and developing event-specific strategies, thereby ensuring more effective preparation for the demands of high-performance competition.

Match dynamics at the junior level may differ substantially from those observed in elite adult competitions due to differences in physical maturity, technical consistency, tactical decision-making, and competitive experience. Although systematic reviews have reported differences across events (1, 2), we found no studies focusing on performance differences between junior levels in all events. Understanding these distinctions is essential for coaches, practitioners, and federations aiming to optimize training strategies and long-term athlete development.

This study aims to address these gaps by analyzing the temporal structure of matches from the 28th Pan-American Junior Badminton Championships held in 2019 across all five badminton events and using observational data. Considering that the temporal structure of matches is different at the adult level, it is expected that events will be different for match duration, real time played, % of real time played, rally time, rest time per rally, total points played, shot per rally, shot frequency, and work density.

## 2 Materials and methods

### 2.1 Sample

Data were collected from the 28th Pan-American Junior Badminton Championships (PanJr 2019). The sample included 124 matches, comprising 277 games and 9,847 rallies across five event types: men's singles (MS), women's singles (WS), men's doubles (MD), women's doubles (WD), and mixed doubles (XD). Athletes competed in the U13, U15, U17, and U19 age categories.

Only matches from the quarterfinal stage onward were included. Nine of the 133 matches were unavailable on the Badminton Pan Am Confederation channel. All matches followed the official rally-point scoring system, consisting of best-of-three games to 21 points. A total of 18 rallies (0.18%) were excluded due to video interruptions, visual obstructions, or technical issues that prevented reliable data collection.

The match recordings were obtained from the official channel of the Badminton Pan American Confederation on the YouTube™ video-sharing platform, which provides free access to the competition's matches. The conducted research is not related to either human or animal use.

### 2.2 Data collection

Match footage was obtained from the official YouTube™ channel of the Badminton Pan Am Confederation. Videos were downloaded using aTube Catcher® (v3.8.9325), identified through the official Badminton World Federation (BWF) Tournament Software®, and systematically organized by age category, event type, and competition stage. An observational system named Ideal Performance® was developed specifically for this study through a partnership between the Federal University of Mato Grosso (UFMT), the Brazilian Badminton Confederation (CBBd), and Ideal Performance®. The tool was implemented in Microsoft Office Excel®.

The inclusion of variables following an analytical sequence, the internal computational procedures, and the validation of the observational system were all conducted prior to its finalization. This process was consensually performed by a system programmer, the principal researcher with 3 years of practical experience in badminton, and an experienced coach with 13 years of practice; all held degrees in Physical Education.

Each match was coded systematically using video analysis, capturing the variables. Rally durations, transitions between rallies, and rest intervals were automatically calculated based on timestamp annotations. To improve coding accuracy, a 50-inch AOC® external LCD monitor was connected via HDMI to a Dell® laptop running Windows 7®. Videos were reviewed frame by frame using Media Player Classic—Home Cinema® (v1.9.18), with court zone diagrams displayed alongside for spatial reference.

Observer reliability was assessed on a randomly selected 10% subsample of matches, in accordance with the minimum percentage determined by Tabachnick and Fidell (21). Intra-observer agreement was established through reanalysis by the primary researcher, while inter-observer agreement was assessed by a second trained observer with 7 years of coaching experience. Intraclass correlation coefficient (ICC) was used for quantitative variables, yielding values for rally time (0.99 and 0.96), rest time per rally (1.00 and 0.99), and shots per rally (1.00 and 1.00), confirming excellent intra- and inter-observer reliability, respectively. Because the remaining variables were dependent on these three, the ICC was not calculated for them.

### 2.3 Variables

This investigation followed two comprehensive analyses of match characteristics in elite badminton, which guided the structuring of our dataset and the selection of variables (15, 22), ensuring consistency with current standards in badminton performance analysis.

The match was adopted as the unit of analysis. This approach enables standardized comparisons across events and matches, avoiding bias that may arise from analyzing data at the match level, where the number of games varies (e.g., two- or three-game matches). Previous studies have reported no significant differences in notational structure between games within the same match (4), supporting this methodological choice. Dependent quantitative variables were selected based on prior literature (4, 15, 22), focusing on the temporal structure of play. The independent categorical variable was event type (MS, WS, MD, WD, XD). The quantitative variables are defined as follows:

- Match duration (min): total time from the first serve until the end of the final rally, including rest periods and the pause at point 11
- Real time played (min): sum of all rally times within the match
- % of real time played: real time played divided by match duration, multiplied by 100
- Rally time (s): time from the service until the shuttlecock touches the ground in a rally
- Rest time per rally (s): time between the end of one rally and the start of the next
- Total points played: number of points played by both players in a match
- Shots per rally: total number of shuttle contacts during a single rally
- Shot frequency (shots·s^−1^): total number of shots divided by real time played
- Work density: ratio between rally time and rest time

### 2.4 Statistical analysis

All procedures were conducted in Python (version 3.11.13, Python Software Foundation), using the libraries pandas, SciPy, stats models, pingouin, and scikit-posthocs. Descriptive statistics are reported as mean (M) and standard deviation (SD). The significance level was set at *α* = 0.05. Before hypothesis testing, the assumptions of normality and homogeneity of variances were tested for each variable within event groups using the Shapiro–Wilk test and Levene's test, respectively.

When both assumptions were met (% of real time played, rally time, rest time, shots per rally, and work density), a one-way ANOVA was applied, with test statistics reported as *F*(df₁, df₂), and effect sizes (ES) calculated as Cohen's *f*—interpreted as negligible (<0.10), small (0.10–0.24), medium (0.25–0.39), and large (≥0.40). Pairwise comparisons were performed using Tukey's HSD, which inherently controls for family-wise error.

For variables that violated the normality assumption (match duration, real time played, total points played, shot frequency), the non-parametric Kruskal–Wallis test was applied, with results reported as *H*(df). In these cases, pairwise comparisons were conducted using Dunn's test with Bonferroni correction. Effect sizes for non-parametric tests were not estimated, since the adopted workflow does not compute Cohen's *f* or *η*² for Kruskal–Wallis.

## 3 Results

Table 1 shows the distribution of two- and three-game matches across the five events in the 2019 Pan-American Junior Championships. Overall, most matches were decided in just two games (76.6% of the total), while a smaller share required three games (23.4%). This pattern was consistent across almost all events, with WD (91.3%) and MD (78.3%) standing out, as nearly all matches were resolved in two games, suggesting greater competitive imbalance in these events.

**Table 1:** Number of two- and three-game matches played in the 2019 PanJr championships by event.

Matches	MS	WS	MD	WD	XD	Total
All matches	27	27	23	23	24	124
Two-game matches	21 (77.8%)	18 (66.7%)	18 (78.3%)	21 (91.3%)	17 (70.8%)	95 (76.6%)
Three-game matches	6 (22.2%)	9 (33.3%)	5 (21.7%)	2 (8.7%)	7 (29.2%)	29 (23.4%)

Men's single (MS), women's single (WS), men's double (MD), women's double (WD), and mixed double (XD).

Table 2 shows the match duration and total points by event in PanJr 2019. Significant differences in match duration were found across events [*F*(4, 119) = 2.526, *p* = 0.044, *f* = 0.291], with WS having the longest duration (31.15 ± 9.52 min), followed by MS (27.69 ± 9.81 min). Doubles events were shorter (23.67–27.43 min), likely due to faster, more offensive play enabled by reduced court space.

**Table 2:** Match duration and total points played in the PanJr 2019 as a function of the event.

Matches		MS	WS	MD	WD	XD	Total	ANOVA (*p*, *f*)
All matches	Matches *n* =	27	27	23	23	24	124	
	Match duration (min)	27.69 ± 9.81	31.15 ± 9.52	25.42 ± 8.62	23.67 ± 6.50	27.43 ± 9.00	27.23 ± 9.05	***p*** **=** **0.044**, *f* = 0.291
	Total points played	78.22 ± 20.25	84.15 ± 21.15	80.30 ± 20.71	69.26 ± 14.09	84.08 ± 20.69	79.37 ± 20.06	*p* = 0.063, *f* = 0.278
Matches		MS	WS	MD	WD	XD	Total	ANOVA (*p*, *f*)
Two-game matches	Matches *n* =	18	21	21	18	17	95	
	Match duration (min)	23.19 ± 4.13	25.81 ± 5.90	21.30 ± 2.31	22.17 ± 4.20	22.63 ± 3.54	23.00 ± 4.36	*p* = **0.022**, *f* = 0.367
	Total points played	68.10 ± 6.66	70.44 ± 8.82	70.28 ± 6.31	65.57 ± 7.18	71.59 ± 6.17	69.02 ± 7.27	*p* = 0.074, *f* = 0.313
Matches		MS	WS	MD	WD	XD	Total	ANOVA (*p*, *f*)
Three-game matches	Matches *n* =	9	6	2	5	7	29	
	Match duration (min)	43.41 ± 7.14	41.83 ± 5.33	40.26 ± 5.85	39.48 ± 5.23	39.07 ± 7.32	41.06 ± 6.10	*p* = 0.763, *f* = 0.277
	Total points played	113.67 ± 4.13	111.56 ± 4.64	116.40 ± 10.41	108.00 ± 7.07	114.43 ± 4.83	113.28 ± 6.04	*p* = 0.446, *f* = 0.401

Men's single (MS), women's single (WS), men's double (MD), women's double (WD), and mixed double (XD).

In the two-game matches, differences remained significant [*F*(4, 90) = 3.027, *p* = 0.022, *f* = 0.367]. MS averaged 23.19 ± 4.13 min, longer than WD (22.17 ± 4.20) and XD (22.63 ± 3.54). Effect sizes suggest meaningful variability due to rally patterns and point structure. In the three-game matches, durations were similar across events [*F*(4, 24) = 0.461, *p* = 0.763, *f* = 0.277], ranging from 39.07 to 43.41 min, likely due to similar demands during extended play.

A similar trend was observed for total points. Across all matches, differences were marginal [*F*(4, 119) = 2.302, *p* = 0.063, *f* = 0.278], with WS averaging the most (84.15 ± 21.15). In the two-game matches, point totals did not differ significantly [*F*(4, 90) = 2.211, *p* = 0.074, *f* = 0.313], and XD had the highest average (71.59 ± 6.17). In the three-game matches, point totals were statistically similar [*F*(4, 24) = 0.963, *p* = 0.446, *f* = 0.401], ranging from 108 to 116, suggesting a plateau once matches reach a third game.

Overall, match duration and total points are influenced by the event and match format. In the two-game matches, singles (WS and MS) are more demanding in terms of time and scoring. In the three-game matches, demands tend to level across events.

Table 3 presents the various match statistics across the five badminton events in the 2019 Pan-American Junior Championships. Overall, match durations varied by event, with WS showing the longest matches on average (31.15 ± 9.52 min) and WD the shortest (23.67 ± 6.50 min). Similarly, real time played followed a comparable pattern, with WS and MS having the highest values (10.26 ± 2.78 min and 9.09 ± 2.90 min**,** respectively), indicating that singles matches generally involve longer active play periods than doubles.

**Table 3:** Comparative analysis of the temporal structure of matches across different badminton events—PanJr 2019.

Statistic	MS (a)	WS (b)	MD (c)	WD (d)	XD (e)	Total	*p*	Cohen's *f*	ES	*Post hoc* differences
Match duration (min)	27.69 ± 9.81	31.15 ± 9.52^d^	25.42 ± 8.62	23.67 ± 6.50^b^	27.43 ± 9.00	27.23 ± 9.05	**0**.**022**	–	–	WS > WD
Real time played (min)	9.09 ± 2.9^c^	10.26 ± 2.78^c,d,e^	6.81 ± 2.37^a,b^	7.55 ± 2.15^^b^^	7.94 ± 2.73^b^	8.41 ± 2.85	**0**.**000**	–	–	WS > MD, WD, XD; MS > MD
% of real time played	33.17 ± 2.74^c,e^	33.37 ± 2.87^c,e^	26.89 ± 2.85^a,b,c^	31.97 ± 3.18^c,e^	28.85 ± 2.63^a,b,c^	30.99 ± 3.79	**0**.**000**	0.907	large	MS, WS > MD, XD; WD > MD, XD
Rally time (s)	6.91 ± 0.92^c,e^	7.32 ± 0.83^c,d,e^	5.03 ± 0.64^a,b,c^	6.48 ± 0.83^b,c,e^	5.58 ± 0.85^a,b,c^	6.31 ± 1.17	**0**.**000**	1.046	large	MS, WS > MD, XD WS > WD WD > MD, XD
Rest time (s)	13.99 ± 2.14	14.79 ± 2.65	13.71 ± 1.48	13.87 ± 2.07	13.78 ± 1.97	14.05 ± 2.12	0.351	0.194	small	No significant difference
Total points played	78.22 ± 20.25	84.15 ± 21.15	80.30 ± 20.71	69.26 ± 14.09^e^	84.08 ± 20.69^d^	79.37 ± 20.06	**0**.**017**	–	–	WS > WD
Shots per rally	6.86 ± 1.17^d^	6.21 ± 0.84^d,e^	6.48 ± 0.90^d^	7.69 ± 1.08^a,b,c^	7.12 ± 1.21^b^	6.85 ± 1.15	**0**.**000**	0.496	large	WD > MS, WS, MD XD > WS
Shot frequency	0.99 ± 0.07^c,d,e^	0.85 ± 0.04^c,d,e^	1.29 ± 0.10^a,b^	1.19 ± 0.06^a,b^	1.27 ± 0.06^a,b^	1.11 ± 0.19	**0**.**000**	–	–	MD, XD, WD > MS, WS
Work density	0.50 ± 0.06^c,e^	0.50 ± 0.06^c,e^	0.37 ± 0.05^a,b,d^	0.47 ± 0.07	0.41 ± 0.05^c,e^	0.45 ± 0.08	**0**.**000**	0.876	large	WS, WD, MS > MD, XD

Men's single (MS), women's single (WS), men's double (MD), women's double (WD), and mixed double (XD).

Note: The letters a–e each represent an event in the first row of each column. When they appear in other rows and columns, it means that the matches from one event are different from those of another event.

The percentage of real time played also differed among events, being higher in WS, MS, and WD (approximately 33%), and lower in MD and XD (approximately 27%–29%), reflecting event-specific pacing and rest patterns. Average rally time ranged from 5.03 ± 0.64 s in MD to 7.32 ± 0.83 s in WS, suggesting that rallies in singles tend to be longer and potentially more physically demanding, whereas doubles may involve shorter but more frequent exchanges. Rest time between rallies was relatively similar across events (approximately 13–14 s), indicating consistent recovery intervals during matches.

Regarding match intensity, shot frequency and work density varied across events. MD and XD presented the highest shot frequency (1.29 ± 0.10 and 1.27 ± 0.06 shots/s, respectively), whereas WS had the lowest (0.85 ± 0.04 shots/s). Work density showed a similar trend, with MD and XD displaying lower values (0.37 ± 0.05 and 0.41 ± 0.05) compared with MS, WS, and WD (approximately 0.50), indicating that doubles events concentrate more shots per unit of time despite shorter rallies.

**Figure 1:**
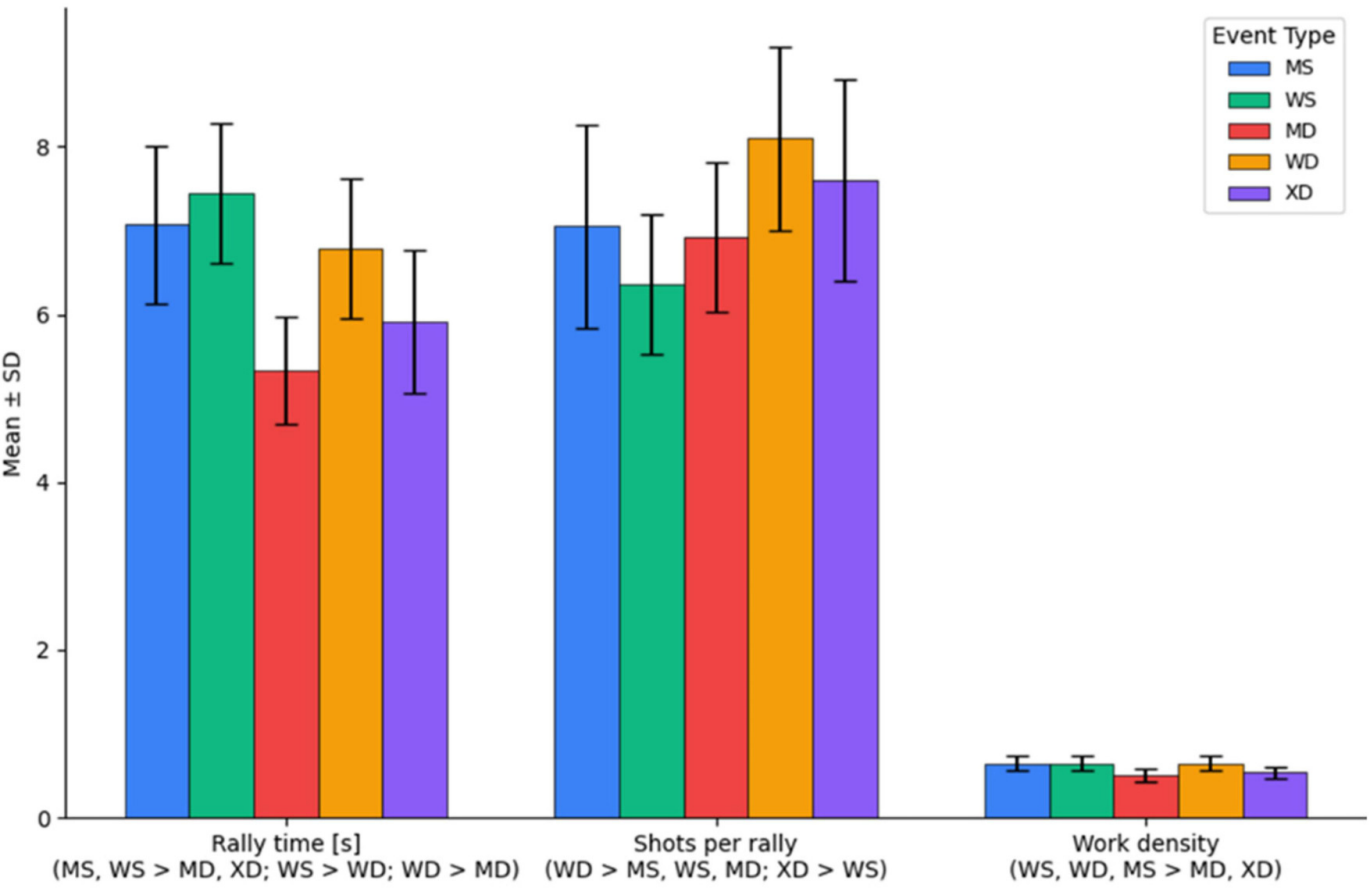
Temporal structure variables of matches in badminton events at the PanJr 2019.

Figure 1 illustrates the mean values of the variables rally time, shots per rally, and work density in the matches, as well as the significant differences among the five events at the PanJr 2019.

The total points played per match were highest in WS (84.15 ± 21.15) and lowest in WD (69.26 ± 14.09), while the number of shots per rally was highest in WD (7.69 ± 1.08) and lowest in WS (6.21 ± 0.84), suggesting differing tactical structures and rally lengths across events.

## 4 Discussion

This study displaying the temporal structure of matches from the 28th Pan-American across all five badminton events had a hypothesis that events would be different for match duration, real time played, % of real time played, rally time, rest time per rally, total points played, shots per rally, shot frequency, and work density. This hypothesis was confirmed for all variables except rest time per rally.

The overall distribution of games that ended in two- or three-game matches in PanJr (three games = 23.4%; two games = 76.6%) was nearly similar to research with elite-level matches from the 2018 World Championships (three games = 23.6%; two games = 76.4%) (15), suggesting comparable levels of balance across junior and senior contexts.

Regarding total match duration, elite-level matches (15) (42.49 ± 14.44 min) are notably longer than those observed in the present study (Table 2) (27.23 ± 9.05 min), both in two-game and three-game matches. This likely reflects superior physical capacity, tactical control, and pacing in adult players.

When analyzing by event-specific and including all matches finished in two or three games, WS and MS recorded the longest match duration, while WD consistently showed the shortest durations, with statistical significance observed only for WS. On the other hand, match duration was not statistically different between singles and doubles in elite-level matches, with only WD being longer than XD (1, 15).

The real time played showed the highest values for singles matches (WS and MS) than for doubles, with the same occurring in elite-level matches (MS, WS > MD and WS > XD) (15), indicating that they generally involve longer active play periods than doubles. In the U19 matches, no difference was found between singles events (16), as in the present study.

The percentage of real time played was also higher in WS and MS and lower in MD and XD, corroborating results obtained with elite athletes from the European Championships in 2016 (14). In the study by Hoffmann et al. (15), singles matches (WS and MS) were higher than MD and XD, and WD was higher than XD in this same variable. In the study by Winata et al. (1), no significant differences were found between the events.

The rally time in singles, with no difference between sexes here, tends to be longer and potentially more physically demanding, whereas doubles (MD and XD) may involve shorter but more frequent exchanges. This same pattern was observed in elite-level matches (MS and WS > MD, XD) (15), and in the study by Winata et al. (1), singles matches also had longer rally durations than all doubles events. In the U19 singles matches, men outperformed women (16) and were inferior to elite-level players (18).

Gomez et al. (5) observed an increase in longer rallies in singles matches at the elite level as well. Their analysis of 60 matches from the 2015 World Super Series and World Championship showed that rally duration affects both physical and cognitive load. In badminton, perception, attention, and decision-making are crucial for adapting to rapid shuttle movements, opponent behavior, and court positioning.

The specific differences observed between events at the junior level reinforce that event-specific demands emerge consistently across competitive levels. Rally time analysis confirmed this trend. WS had the longest rallies, while MD and XD had shorter ones. This reflects the more offensive playing style in MD and XD, where higher shuttle speed and aggressive strokes lead to quicker point resolution. In contrast, WD rallies last longer due to a balance between moderate pace and high defensive capacity, which creates more sustained exchanges.

Rest time between rallies was similar across events, indicating consistent recovery intervals during matches. This same pattern was observed in elite-level matches (15). Although at the 2016 European Championship, XD had longer rest times than WD (4), and at the London Olympics, MD had longer rest times than WD. However, such differences were not observed in Beijing or Rio (4).

In another comparison, MS had longer rest times than WS at the Beijing Olympics (3). In the study by Le Mansec et al. (14), WD was lower than XD, but neither differed from singles matches, and in the study by Winata et al. (1), men were superior to women in both singles and doubles. In the U19 singles matches, men outperformed women (16) and were inferior to elite-level players (18).

These results show that junior players do not make effective use of this interval compared with elite players, who have averages of 22.96 s (15) vs. 14.05 s. Even when players have already recovered from a rally—the primary function of this interval—this moment is also useful for making strategic adjustments during the match. As a result, these low rest time values produced much higher % real time played than what we typically see in elite-level matches.

The total points played were highest in WS and lowest in WD, contradicting the matches from the 2018 World Championships, with no event-specific differences in the average total points (15).

The shots per rally were highest in WD and lowest in WS. In elite-level studies, the same result (WD > MS, WS, MD) was found in matches from the 2018 World Championships (15). However, in the systematic review by Winata et al. (1), no differences were found between singles and doubles, unlike what was found here. In singles matches, men executed more shots per rally than women in the elite-level1 and U19 (16). When comparing MS at the elite level and U19 levels, Leong and Krasilshchikov (16) found that the number of shots per rally was higher for the elite-level players.

Shot frequency was higher for doubles, whereas WS had the lowest, and men's singles were superior to women’s singles. In the U19 singles matches, men were equal to women (16), and the same occurred in the present study, but at the elite level, men were superior to women in both singles and doubles, and no differences were found between doubles and singles matches (1). Maintaining the discussion with elite-level matches, this same pattern found here was observed in the 2018 World Championships (15). The similarities found in shots per rally and shot frequency between junior and elite-level players in the 2018 World Championships reflect behaviors that occur within each event.

In work density, MD and XD display lower values compared with MS, WS, and WD, indicating that those events involve higher shot frequency, shorter rallies, and more concentrated activity. The elite-level matches showed a similar trend for singles superiority (1, 15). This event-specific variation underscores the importance of tailoring training strategies to the unique demands of each event, emphasizing endurance and pacing for singles and speed, coordination, and teamwork for doubles.

As we see, higher averages in intensity variables (rally time, shots per rally, and work density) at elite-level (1, 4, 11, 15, 22), differences between levels may also reflect players’ ability to recognize and act on relevant cues. According to Chen et al. (23), elite players have better reaction time, perceptual accuracy, and attention control than amateur athletes. They focus more effectively on relevant information, while less experienced players are more prone to distraction. Xiong and Song (24) also observed that experienced athletes are better at making decisions and locating useful visual information during play.

The results of the present study have several practical implications for coaches, sport scientists, and federations engaged in junior athlete development. In doubles, the clear differences across events indicate that training programs should be tailored to the technical and temporal demands of each modality, even at the junior level. For instance, players in men's and mixed doubles should focus on serve-return dynamics and high-intensity, short-duration rallies. Women's double players may benefit from endurance-oriented training and drills aimed at increasing rally length and shot volume. Defensive shot training is also essential in this event, as the ability to stay in the rally is a key factor.

Although this study provides new insights into the temporal demands of badminton across all five events in junior international competition, some limitations must be acknowledged. The analysis was based on data from a single tournament, which may limit the generalizability of the results to other junior competitions. Second, only video footage from official match broadcasts was used for data collection. While this ensured access to high-level matches, it introduced constraints such as occasional changes in camera angles and incomplete coverage of certain rallies.

We suggest that more studies be conducted with juniors, especially in relevant competitions, such as the one used here, rather than conducting studies with simulated matches. Furthermore, studies comparing existing categories at the junior level are needed.

## 5 Conclusion

This study offers a comprehensive analysis of temporal characteristics in junior badminton across all five events. The results showed that temporal structure is different between events, except for the rest time per rally. Although event-specific patterns seen at the elite level are already present among juniors, younger athletes tend to have shorter rallies, lower work density, and slightly reduced technical performance. These findings reinforce the importance of developmentally appropriate and event-specific training approaches to prepare junior athletes for the physical and tactical demands of elite badminton.

## Data Availability

The original contributions presented in the study are included in the article/Supplementary Material; further inquiries can be directed to the corresponding author.
